# Temporally stable adaptation is robust, incomplete and specific

**DOI:** 10.1111/ejn.13355

**Published:** 2016-08-19

**Authors:** Katinka van der Kooij, Krista E. Overvliet, Jeroen B. J. Smeets

**Affiliations:** ^1^ Department of Human Movement SciencesVrije Universiteitvan der Boechorststraat 91081 BTAmsterdamThe Netherlands; ^2^ Department of Biological Psychology and NeuropsychologyUniversität HamburgHamburgGermany

**Keywords:** retention, sensory realignment, training, visuomotor adaptation

## Abstract

Sensorimotor adaptation, the process that reduces movement errors by learning from sensory feedback, is often studied within a session of about half an hour. Within such a single session, adaptation generally reaches plateau before errors are completely removed. However, adaptation may complete on longer timescales: the slow components of error‐based adaptation are associated with good retention. In this study, we tested how adaptation evolves over time by asking participants to perform six adaptation sessions on different days. In these sessions, participants performed a three‐dimensional reaching task while visual feedback about endpoint errors was rotated around the cyclopean eye. In addition, context specificity of the adaptation was addressed by measuring inter‐limb transfer and transfer to visual and proprioceptive perceptual tasks. We show that from the second session on, the adaptation was retained almost completely across sessions. However, after six learning sessions, adaptation still reached plateau before errors were completely removed. The adaptation was specific: the adaptation did neither transfer to the other hand, nor to the visual, and only marginally to the proprioceptive perceptual estimates. We conclude that motor adaptation is robust, specific and incomplete.

## Introduction

One reason for our ability to make fairly accurate goal‐directed movements in the face of a changing body and world is that the brain adapts the movement plans in response to sensory feedback about errors; a process called sensorimotor adaptation. Sensorimotor adaptation is generally studied using a paradigm in which participants make goal‐directed movements in a perturbed environment that evokes large initial errors that participants need to adapt to. The perturbation may be induced by applying forces to the hand (e.g. Shadmehr & Mussa‐Ivaldi, 1994; Smith *et al*., 2006) or by rotating visual feedback (e.g. Cohen, 1967; Redding & Wallace, 1988; Fernandez‐Ruiz *et al*., 2004; Mazzoni & Krakauer, 2006; Burge *et al*., 2008; Cressman & Henriques, 2009; van der Kooij *et al*., 2013; van der Kooij & Overvliet, 2016). When visual feedback about errors is available, participants learn by correcting for a fraction of the error on each trial. However, when error‐feedback is no longer available, errors drift back towards baseline showing that retention is incomplete (e.g. Choe & Welch, 1974; Smith *et al*., 2006; Cheng & Sabes, 2007). Clearly, adaptation to consistent changes should be temporally stable, whereas adaptation to transient changes such as fatigue should not. A dual‐rate state‐space model shows exactly such behaviour: it consists of a fast process that learns rapidly from performance errors but has poor retention and is therefore temporally labile, and a slow process that learns slowly from the performance errors and has good retention and is therefore temporally stabile (Smith *et al*., 2006). According to this model, the retention of adaptation across sessions should be based on the slow process (Joiner & Smith, 2008). However, according to the dual‐rate model, adaptation can only complete when retention of one of the two processes is perfect. Although retention of the slow process was originally considered very good but imperfect (Smith *et al*., 2006), insufficient long‐term studies have been performed to reliably determine whether adaptation completes over time. Moreover, recent research on motor adaptation has shown that multiple mechanisms contribute to the temporally stable component of adaptation, some of which are associated with very good retention (Huberdeau *et al*., 2015). Especially later on in adaptation, when the lion's share of errors has been reduced, reinforcement learning and use‐dependent plasticity may become more important (Huberdeau *et al*., 2015). In addition over time, sensory realignment may add to the visuomotor adaptation by changing the mapping between vision and proprioception (Zbib *et al*., 2016). Hence, even if retention of the slow process is incomplete, it may be possible that additional mechanisms complete the temporally stable adaptation over time.

The aim of this study was to investigate how learning and retention of adaptation evolve over a time‐scale of days rather than minutes. On six different days, participants performed 20‐minute sessions of a three‐dimensional reaching task in which a rotational perturbation was imposed on visual feedback about endpoint errors.

## Methods

### Participants

A within‐participants design was used in which twelve participants took part (age 24.0 ± 3.5; mean ± standard deviation), either voluntarily (*N* = 2, employees of the Vrije Universiteit) or reimbursed €8 per hour (*N* = 10, students at the Vrije Universiteit). One participant dropped out after the first session and was eliminated from the data set. Participants were right handed according to the Edinburgh handedness test and had good stereo acuity as tested with the Randot StereoFly test (median stereo acuity of 40 seconds of arc). Participants were naive to the purposes of the experiment. The total duration of the experiment for a participant was about 4.5 h.

### Set‐up

**Figure 1:**
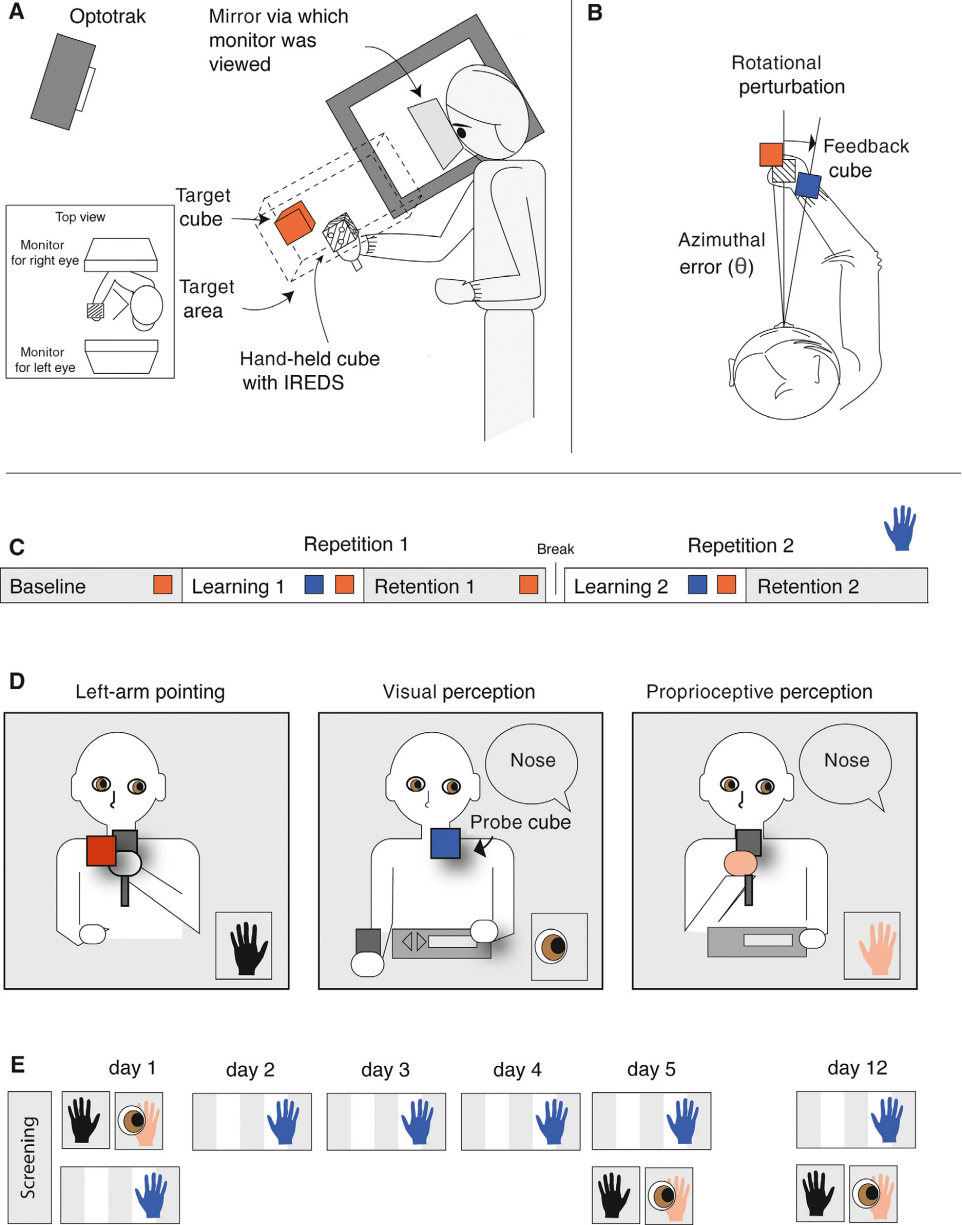
Methods. (A) Experimental set‐up. (B) Rotational perturbation imposed on the visual feedback. (C) Adaptation phases: in each session subjects performed five alternating phases of 50 trials without and with feedback. (D) Transfer tests: pointing with the left arm, visual and proprioceptive perception of egocentric locations. (E) Temporal in the order of adaptation phases and transfer tests. Symbols correspond to the ones in (C and D): blue right hand for pointing, left hand for left‐hand pointing, right hand and eye for proprioceptive and visual perception of egocentric locations respectively.

The set‐up was similar to the one used in earlier studies (van der Kooij *et al*., 2013, 2015) and is described below. Participants were seated in a light proof room, where they viewed two separate CRT displays (48 × 31 cm; viewing distance about 40 cm; resolution 1096 × 686 pixels, 160 Hz), one with each eye via mirrors (Fig. 1A). Infra‐red emitting diodes (IREDs) were mounted on a cube with 5‐cm edges with a handle that participants held in their hand and that allowed us to track the movements of the participants' hand at 100 Hz with an Optotrak 3020^®^ motion analysis system (NDI, Waterloo, ON, Canada). To be able to render an adequate image of the 3D scene without having to restrain the participant's head, IREDs were mounted on a bite board that participants held in their mouth and that was not connected to the set‐up. For each participant, we determined the eyes' locations relative to the bite‐board in a calibration session (Sousa *et al*., 2010). This allowed us to render an appropriate new image of the 3D scene for each eye with a latency of ~25 ms between participants' movements and the corresponding update of the display. In this way, all cues for an object's position in depth (target vergence, image size, motion parallax, etc.) except for accommodation vary consistently with the simulated distance. As a result, this setup renders a realistic representation of the 3D space in front of the participant, without them being able to see their hand or the physical cube as they move from target to target.

### Pointing task

A trial started with the appearance of a red target cube at a random location within a 10 by 10 by 30 cm target area elongated along the participants' line of sight with the centre at 40 cm distance (Fig. 1A). As soon as the target had appeared, the participant could initiate a pointing movement with the right arm. A movement endpoint was defined by a velocity of less than 2 cm/s for a duration of 300 ms. At this point, we either provided visual feedback on the position of the cube at the end of the movement or presented the next target, depending on the adaptation phase. In the trials with visual feedback, this feedback was provided by means of a blue feedback cube that was presented for 500 ms. To induce a perturbation that participants had to adapt to, the tracked 3D position of the hand‐held cube was rotated azimuthally 10° leftward around the cyclopean eye before rendering the feedback cube based on this position. This way, the movement endpoint had to be 10° rightward of the target for the participant to see the target and feedback cube aligned. As the azimuthal rotation was performed on the 3D scene, and most depth cues depend on the azimuth, the rotation resulted in minor changes in depth cues despite not changing the simulated egocentric distance.

Alternating phases of 50 trials without any visual feedback and with visual endpoint feedback formed three adaptation phases (Fig. 1C). In an initial ‘baseline’ phase without visual feedback, we measured baseline biases. In a subsequent ‘learning’ phase, we provided (perturbed) visual feedback and measured learning, whereas in a following ‘retention’ phase without visual feedback, we measured retention of the adaptation. After the participants had performed each phase, there was a short break to prevent arm fatigue. After that, there was one repetition of the learning and retention phase such that savings – faster re‐learning upon second exposure the perturbation – could be assessed.

### Transfer tasks

To test specificity of the adaptation we tested both inter‐limb transfer and transfer to two perceptual tasks (Fig. 1D). Inter‐limb transfer was tested by asking participants to hold the cube in the left hand and align it to 100 visual targets appearing one by one in the same way as the targets in the adaptation task, without receiving visual feedback on the responses. This was tested on day 5 and 12, and compared with a baseline measurement before the adaptation task on day 1.

We tested whether the visuomotor adaptation transferred to visual and proprioceptive perception of egocentric positions by asking subjects to position either a visual probe cube or their hand straight ahead of a part of their body. These perception trials started with a voice cue indicating which body part was tested: the left shoulder, nose or right shoulder. In the visual perception trials, participants could move the visual probe cube using the left and right arrows on a keyboard for horizontal displacement and up and down arrows for vertical displacement (Fig. 1D, centre panel). In the proprioceptive trials, participants moved the hand‐held cube with their right arm to a point straight ahead of the required body part. In both visual and proprioceptive perception trials pressing the spacebar on the keyboard triggered the registration of the judged position. Visual and proprioceptive trials were interleaved and each position was replicated ten times for each position, but only the estimates of the position straight ahead of the nose were used in the data analysis.

### Procedure

The experimental sessions are schematically drawn in Fig. 1E. On the screening day (which was performed within 5 days before the start of the experiment), we explained the experimental procedures after which participants gave written informed consent. We conducted the Randot StereoFly test and we calibrated the participants' bite board that was used in the virtual reality (VR) set‐up. Afterwards, we administered 100 trials of the right‐arm pointing task. This was done without feedback, so that we could check participants' natural bias, defined as the average distance from the target. A large natural bias will interfere with the learning, so we checked for each participant whether the natural azimuthal bias of a participant was less than 5° (50% of the size of the perturbation). None of the participants had a large natural bias (minimum −0.78°, maximum 1.16°), and thus no participants were excluded. The screening session took in total about 30 min.

On day 1, participants first performed the baseline measures of left‐arm pointing, followed by the baseline measures of the perceptual tasks and the first adaptation session taking about 45 min. On day 2, day 3 and day 4 participants performed only the five adaptation phases, taking about 20 min each day. On day 5 participants first performed the five adaptation phases, after which we started the first post‐test of left‐arm pointing and the perceptual tasks. Each test (adaptation, left‐arm pointing, perceptual) was separated by a break of a minimum of 2 min (depending on the preference of the participant). In total, this session took about 45 min. One week later (day 12), participants came back to perform the last session, consisting of the five adaptation phases directly followed by the second post‐tests of pointing with the left arm and the perceptual tasks, again taking about 45 min.

### Data analysis

As we imposed a perturbation in the azimuthal direction, we analyzed azimuthal error (θ), which was defined as the difference in the azimuthal direction of the tracked position of the hand‐held cube and visual target. As one of the aims of this study is to test whether processes that are additional to the dual‐rate model complete adaptation over time, we use model‐free adaptation parameters that can capture components of temporally labile and stabile adaptation that are not described by the dual‐rate model. We used four model‐free parameters: the baseline bias, learning asymptote, retention asymptote and savings. We denote a movement towards a single target as a target and a trial and the baseline bias is defined as the mean azimuthal error in all trials of the baseline phase. The amount of adaptation was analyzed by the learning asymptote**,** which is defined as the mean azimuthal error in the last 25 trials of a learning phase. The temporally stable adaptation is quantified by the retention asymptote, defined as the mean azimuthal error in the last 25 trials of a retention phase. We estimated the learning rate *B*_r*i*_ in repetition *i* by the mean adaptation in the second and third trial of a learning phase relative to the previous phase without feedback:

$$B_{r1}=\bar{\theta_{t52:t53}}-\text{baseline}\;{\text{bias}}_{r1}$$

$$B_{r2}=\bar{\theta_{t152:t153}}-\text{retention}\;{\text{asymptote}}_{r1}$$

Savings is hence defined as the learning rate in repetition 2 minus the learning rate in repetition 1

$$\text{Savings}=B_{r2}-B_{r1}$$

Inter‐limb transfer was assessed by the azimuthal error in the left‐arm pointing task Perceptual transfer was measured from (the azimuthal component of) the judged position straight ahead of the nose in the perceptual trials. The judged position straight ahead of the left and right shoulder were not taken into account, because leaving the distance at which participants pointed in the proprioceptive perception trials unconstrained confounded the azimuthal direction by pointing distance.

Data were processed offline using matlab R2015a and statistical analyses were performed using ibm spss version 22.0. The different parameters were tested for normality using Shapiro Wilkinson tests with a significance level of 0.05. As the parameters passed these tests, we used repeated measures of analyses of variance (anova's) to test our hypotheses. The parameters were analyzed in separate repeated‐measures anova's. To test whether the learning and retention asymptotes evolved over time, we performed repeated measures anova's with the factors day and repetition. Whether baseline biases and savings evolved over time was each analyzed with one‐way repeated measures anova's with day as a factor. Whether the adaptation transferred to pointing with the other limb and to the perceptual tasks was assessed by entering the left arm pointing error and the proprioceptive and visual estimates of the direction straight ahead of the nose in repeated measures anova's with day as a within‐subjects factor. *Post hoc t*‐tests were performed with a *P*‐value corrected for multiple comparisons using matlab's ‘mafdr’ function (False Discovery Rate), using the linear step‐up (LSU) procedure introduced by Benjamini & Hochberg (1995).

To test whether the adaptation parameters gradually increased over test‐days or rapidly saturated, we modelled the change in adaptation parameter as a function of session (1–6) by an exponential and a linear model. Both models contained two free parameters (time constant and asymptote for the exponential model and an offset and slope for the linear model). We fitted these models to the mean parameter values using matlab's lsqcurvefit function. A goodness of fit comparison was made using *Χ* ^2^.

In an exploratory analysis, we examined which of the parameters that described adaptation on the first test day best reflected long‐term adaptation. To this end, we assessed the correlation between the retention asymptote, savings and baseline bias at day 1–5 to the final adaptation that was defined as the baseline bias at day 12.

## Results

**Figure 2:**
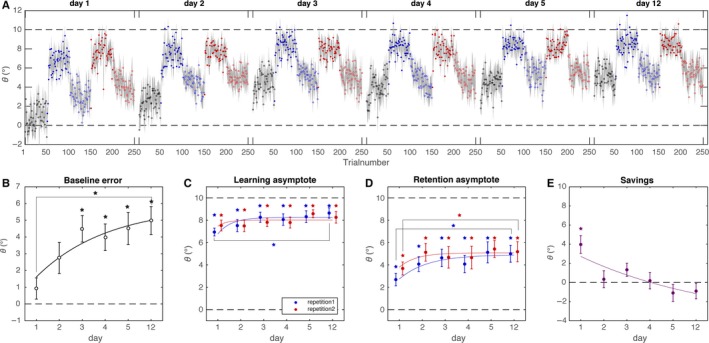
Pointing task. (A) Azimuthal errors averaged over participants as a function of trial number in the different days. Open symbols indicate trials without visual feedback, whereas filled symbols indicate trials with visual feedback. Shaded areas indicate the standard error of the mean. (B) Baseline bias for the different days and exponential fit through the data. (C) Learning asymptotes for the two repetitions of the learning block on each day with exponential fit through the data. (D) Retention asymptotes for the two repetitions of the retention block on each day with exponential fit. (E) Savings as function of day with exponential fit. Error bars represent standard errors of the mean and stars indicate significant differences.

A linear regression of the spherical coordinates of the pointing responses onto the spherical coordinates of the target cubes showed that participants followed the task, basing their pointing responses on the 3D position of the target cubes as the slope of regression was significant in for all dimensions. The slope of the regression was 1.063, 95% CI [1.057, 1.068] for the azimuthal direction, 1.028, 95% CI [1.020, 1.036] for the elevation and 0.780, 95% CI [0.773, 0.788] for distance, indicating that the distance of the target cubes was slightly under estimated. As expected, participants adapted their pointing errors to the perturbed feedback and their errors drifted towards baseline in the retention phases, without returning completely to baseline (Fig. 2A). In the repetition of the experiment on the second and later days, participants seem to have retained most of what they had learnt on the day(s) before. They started at a similar value as where they ended the session at the previous day, and also appeared to end at a higher adaptation value in the learning blocks than on the previous day.

### Baseline bias

The repeated measures anova on the baseline biases revealed a main effect of day (*F*_1,5_ = 4.91, *P* = 0.001, $\eta_{p}^{2}=0.329$), indicating that over days, participants increasingly adapted their baseline bias to correct for the rotational perturbation.

### Learning asymptote

The repeated measures anova on the learning asymptotes also revealed a main effect of day (*F*_5,50_ = 6.016, *P* < 0.001, $\eta_{p}^{2}=0.376$), whereas there was no main effect of repetition (*F*_1,10_ = 0.093, *P* = 0.767, $\eta_{p}^{2}=0.009$) and no interaction of day and repetition *F*_5,50_ = 0.995, *P* = 0.430, $\eta_{p}^{2}=0.09$). This suggests that participants mainly learnt over days and that there was no additional learning between blocks within a single day. *Post hoc t*‐tests with in which we compared the learning asymptote between subsequent days and between the first and final day showed that there was a significant difference between the learning asymptote in the first repetition of the first test day and the learning asymptote on the first repetition of the final test‐day (*t*(10) = −5.034, *P* = 0.006), whereas there were no other significant differences.

Comparing the exponential and linear model for the change in parameter over time showed that the exponential model described the data better (with *Χ* ^2^ = 0.07 for the exponential fit and *Χ* ^2^ = 0.08 for the linear fit), indicating that the adaptation saturated rather than continued to increase. Moreover, the values obtained for the parameter time constant were for the first repetition 0.58 days and for the second repetition 0.37 days (Fig. 2C). This indicates that the adaptation had reached asymptote after a few days.

### Retention asymptote

Similar to the repeated measures anova on the learning asymptote, the anova on the retention asymptotes showed a main effect of day (*F*_5,50_ = 4.57, *P* = 0.002, $\eta_{p}^{2}=0.314$), and no main effect of repetition (*F*_1,10_ = 1.275, *P* = 0.285, $\eta_{p}^{2}=0.113$). However, there was a significant interaction of day and repetition (*F*_5,50_ = 2.52, *P* = 0.04, $\eta_{p}^{2}=0.202$). *Post hoc t*‐tests comparing the retention asymptotes between repetitions of the different days showed no significant differences. *Post hoc t*‐tests comparing the retention on the two repetitions between different days, however, showed that the retention in the first repetition increased from day 1 to day 2 (*t*(10) = −3.26, *P* = 0.043) as did the retention in the second repetition (*t*(10) = −3.21, *P* = 0.046). There were no other significant differences.

In line with our finding for the learning asymptote, the exponential model best described the development of the retention asymptote (with *Χ* ^2^ = 0.12 for the exponential fit and *Χ* ^2^ = 0.37 for the linear fit). Again, the estimated time constants indicated that the retention saturated after a few days (with the time constants for the first and second repetition being 1.23 and 0.73 days respectively).

### Savings

The repeated measures anova on savings revealed a main effect of day (*F*_5,50_ = 4.473, *P* = 0.002, $\eta_{p}^{2}=0.309$). *Post hoc t*‐tests revealed that the savings were significantly greater than zero only on day 1 (*t* = 1.906, *P* = 0.002) but not on later days.

### Transfer

**Figure 3:**
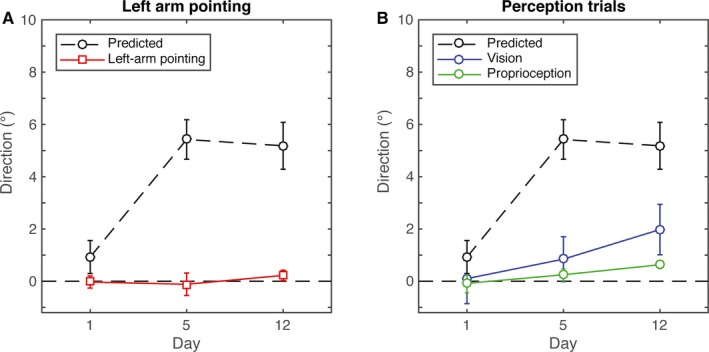
Transfer. (A) Mean azimuthal direction when pointing with the left arm as a function of day. (B) mean azimuthal direction relative to baseline indicated on the visual and proprioceptive perception trials as a function of day. Error bars indicate the standard error of the mean across subjects.

To calculate the change in azimuthal error that would reflect full transfer of adaptation, we used the retention asymptote in repetition 2 of the corresponding day. This showed that full transfer of adaptation would yield a change in azimuthal direction of approximately 5° between day 1 and day 5 or 12 (predicted in Fig. 3A). The transfer is clearly less. The one‐way anova on the left‐arm pointing data showed that there was no main effect of day (*F*_1,11_ = 0.47, *P* = 0.63, $\eta_{p}^{2}=0.05$), indicating that the increased adaptation did not transfer to the untrained arm.

For full transfer to the perception trials (Fig. 3B), the predicted effect is the same as for full transfer to pointing with the left arm. Also here, the observed transfer is clearly less. The repeated measures anova on the visual estimates of the direction straight ahead of the nose showed no effect of day (*F*_2,18_ = 2.283, *P* = 0.131, $\eta_{p}^{2}=0.202$) indicating that adaptation of pointing responses did not transfer to visual estimates of the direction straight ahead of the nose. The repeated measures anova on the proprioceptive estimates of the direction straight ahead of the nose, in contrast, did show a significant effect of day (*F*_2,18_ = 4.857, *P* = 0.021, $\eta_{p}^{2}=0.35$), indicating that proprioceptive estimates shifted in the direction of the adaptation in the pointing task. *Post hoc* paired‐sample tests showed significant differences between day 5 and day 12 (*t*(9) = −2.52, *P* = 0.046) and between day 1 and day 12 (*t*(9) = −2.52, *P* = 0.045 but no significant difference between day 1 and 5.

**Figure 4:**
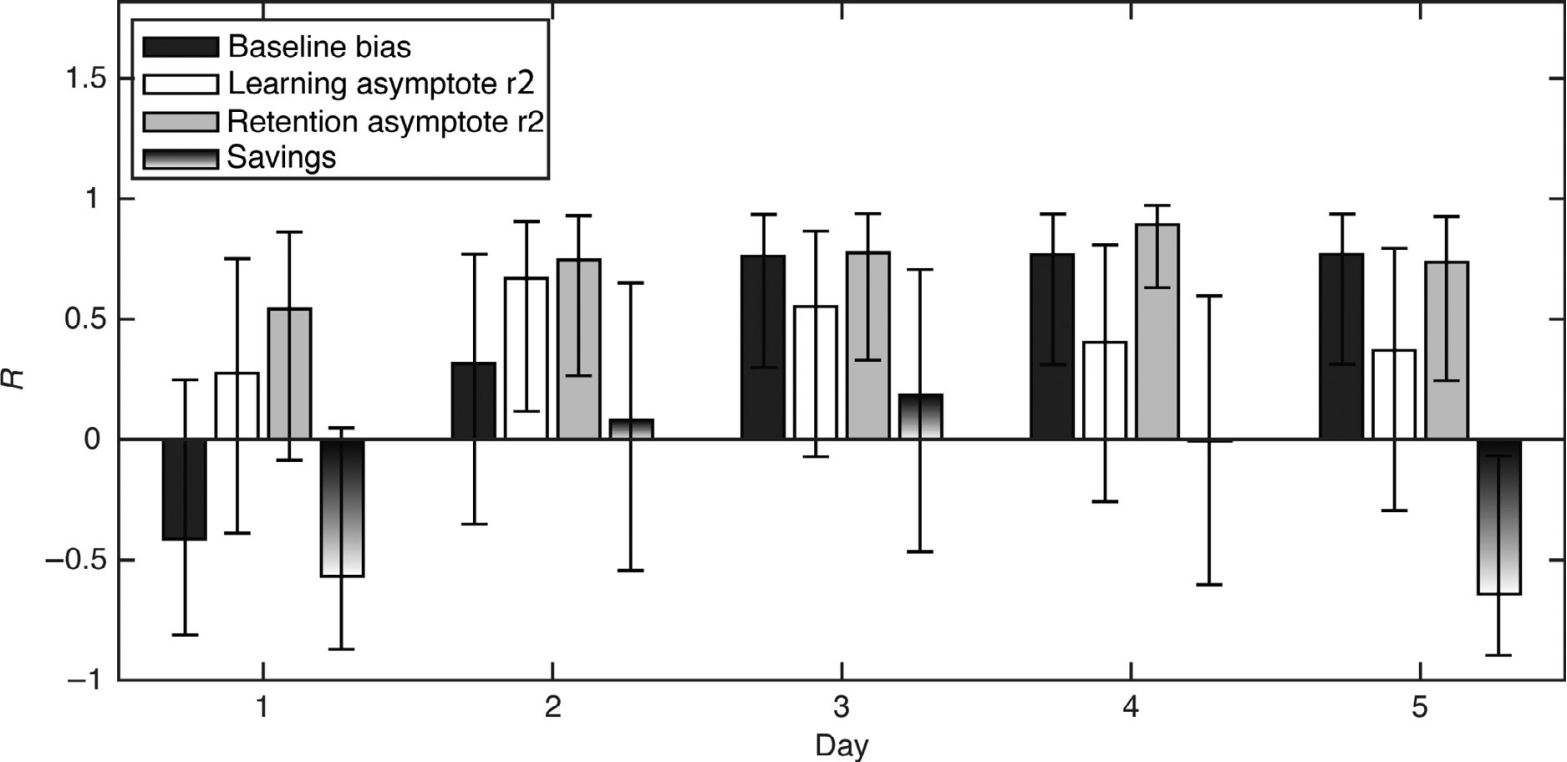
Correlations with baseline bias on the final day. (A) Pearson's *R* for the correlation of the baseline bias, learning asymptote on repetition 2, retention asymptote on repetition 2 and the baseline bias on day 12. Error bars represent the 95% confidence interval on *R* as estimated with matlab's ‘corrcoef’ function.

In the final exploratory analysis, we determined how the different adaptation parameters were related to the final adaptation. To this end, we correlated the baseline bias, learning asymptote, retention asymptote and savings in the different sessions to the final adaptation (baseline performance at the final test day). Figure 4 plots the correlation coefficient *R* with lower and upper limits of the 95% confidence interval.

The retention asymptote showed the clearest pattern of correlation with the baseline bias at the final test day: from day 2 on, it was significantly and positively correlated with the baseline bias at the final test day (*R* = 0.746, *P* = 0.028 for day 2, *R* = 0.776, *P* = 0.026 for day 3, *R* = 0.893, *P* = 0.004 for day 4, *R* = 0.736, *P* = 0.028 for day 5). Moreover, the correlation was relatively constant across days. Baseline biases showed a pattern of correlations similar to the retention asymptote, but were only significantly correlated with the baseline bias at the final test day, from day 3 onwards (*R* = 0.762, *P* = 0.026, for day 3, *R* = 0.768, *P* = 0.026 for day 4 and *R* = 0.768, *P* = 0.026, for day 5). The baseline bias did not show stronger correlations to the baseline performance at day 6 than the retention asymptotes. This suggests that the baseline bias at day *n* was strongly related to the retention asymptote at day *n* + 1, which was confirmed by correlation analyses: (*R* = 0.776, *P* = 0.013 for day 1; *R* = 0.734, *P* = 0.013 for day 2; *R* = 0.475, *P* = 0.14 for day 3, *R* = 0.819, *P* = 0.01, for day 4 and *R* = 0.737, *P* = 0.013 for day 5). The learning asymptote, and savings were not significantly correlated with the baseline bias at the final test day.

## Discussion

In this study, we investigated how adaptation evolves over training episodes repeated on different days. We tested adaptation in a 3D pointing task in which participants learnt to correct for a 10° azimuthal rotation of visual feedback around the cyclopean eye. Alternating phases without and with visual feedback on hand position allowed us to test learning and retention as well as savings. Transfer of the adaptation was assessed by asking participants to use the un‐adapted left arm in the same 3D pointing task (inter‐limb transfer) and by visual and proprioceptive judgments of straight ahead (perceptual transfer). We found that the baseline bias, learning asymptote and retention asymptote all changed in the direction of the rotational perturbation. The adaptation was robust: participants started a session at a baseline level similar to the level at which they had ended the session at the previous day. The adaptation was also specific: we found a marginal transfer to the proprioceptive estimate of straight ahead, but not to the visual estimate of straight ahead or to pointing with the other arm. Both the retention asymptote (from the second day onwards) and the baseline bias (from the third day onwards) were related to the final adaptation. The adaptation was incomplete: even on the final test day, the learning asymptote was lower than the perturbation and thereby our data are consistent with perhaps the most radical prediction of the dual‐rate state space model: the level of adaptation is limited by the balance between learning and forgetting in the slow process (van der Kooij *et al*., 2013). Furthermore, because the learning and retention asymptotes did not increase noticeably over later training episodes, our results do not support the idea that additional learning mechanisms finally complete the adaptation (Huberdeau *et al*., 2015).

One such mechanism that could enhance the adaptation beyond what would be expected based on the dual‐rate model is an increase in the learning rate for repeated errors (Herzfeld *et al*., 2014). Increased learning rates would change the balance between learning and forgetting, allowing the learning asymptote to increase beyond the level that would be predicted based on the learning and forgetting rates for the first day. As the adaptation appeared to saturate (with time constants for the learning asymptote in the first and second repetition being 0.58 and 0.37 days), our results do not support this idea.

Although we did not find that the adaptation asymptote increased over time, we did find that learning rates increased over time as there were savings. Savings have been previously related to explicit processes (Haith *et al*., 2015) and our paradigm may have facilitated explicit processes by providing endpoint feedback rather than continuous feedback (Hinder *et al*., 2008) and by introducing the perturbation suddenly rather than gradually (Michel *et al*., 2007). Savings do not seem to have significantly affected the long‐term adaptation as savings were not related to the final adaptation. Moreover, the adaptation did not generalise to pointing with the other limb whereas explicit processes are associated with good generalisation (Heuer & Hegele, 2011). Consistent with our finding that savings were not related to the final adaptation, explicit processes have been found to add to the adaptation without interfering with implicit processes of adaptation (Mazzoni & Krakauer, 2006). There are also studies that suggest that explicit processes do interfere with implicit adaptation (Michel *et al*., 2007; Hinder *et al*., 2008). Although these studies varied the contribution of explicit processes to the adaptation by varying the feedback (e.g. continuous vs. endpoint feedback), and therefore the effects of explicit processes and feedback may have been confounded.

The fact that the retention asymptote did not change noticeably, appears inconsistent with the idea that the relative contribution of motor adaptation mechanisms that are associated with good retention increases over time (Shmuelof *et al*., 2012; Huberdeau *et al*., 2015). Two such mechanisms are reinforcement learning and use‐dependent plasticity. These mechanisms are held to rely on the repetition of specific movements (e.g. Bütefisch *et al*., 2000; Huang *et al*., 2011) and may not have contributed to the adaptation in our study as each target appeared in a random but reachable direction relative to the previous target, requiring participants to move in a different direction on each trial.

Although letting participants point in different directions while perturbing the visual feedback around the cyclopean eye rather than around a movement's starting position (e.g. Mazzoni & Krakauer, 2006; Cheng & Sabes, 2007; Burge *et al*., 2008; Galea *et al*., 2015) may have prevented persistent processes of motor learning it would have set the condition for realignment of vision and proprioception. Sensory realignment affects all processes using the realigned senses and is therefore measured in generalisation tasks in which participants are for instance asked to make perceptual judgments following a visuomotor adaptation task. The contribution of sensory realignment to adaptation is confirmed by studies that have used such a generalisation paradigm and have shown that adaptation involves both motor and sensory components, with the sensory realignment generally being less than the adaptation of motor commands (Uhlarik & Canon, 1971; Templeton *et al*., 1974; Cressman & Henriques, 2009; Priot *et al*., 2010; van der Kooij *et al*., 2013). However, we found that in addition to being incomplete, the adaptation was also specific: it did not transfer to pointing with the other limb and only marginally to proprioceptive estimates of straight ahead. This indicates that there was only marginal realignment of vision and proprioception. At the same time, the fining that the adaptation was specific is consistent with the idea that sensorimotor adaptation is context‐dependent (e.g. Ingram *et al*., 2013) and that adaptation is generally confined to the perceptual and motor effectors used during exposure (Redding & Wallace, 1988). Moreover, it is also consistent with the finding that the movements of the two arms (Wang & Sainburg, 2003) and even the independent movements of the thumb and index finger (Schot *et al*., 2014) can adapt independently. A number of factors may have contributed to the marginal sensory realignment in our study: the size of errors, contextual information, temporal factors and cue conflict.

First, the size of errors may have affected the perceptual realignment: it has been shown that smaller errors tend to be ascribed to the body – setting the conditions for sensory alignment – whereas lager errors tend to be ascribed to perturbations in the environment (Wei & Kording, 2009). Hence, perturbing the feedback with a ten‐degree rotation may have not led to sensory realignment whereas smaller perturbations would have resulted in realignment. It must be noted however that the ten‐degree perturbation used in this study was smaller than the perturbation used in most prism‐adaptation studies (Cohen, 1967; Choe & Welch, 1974; Bedford, 1993; Fernandez‐Ruiz *et al*., 2004) and also smaller than the 30‐degree rotation used in many visuomotor adaptation studies (Baraduc & Wolpert, 2002; Scheidt & Ghez, 2007; Haith *et al*., 2015).

Second, contextual factors may contribute to the realignment. We found less proprioceptive realignment compared to an earlier study (van der Kooij *et al*., 2013) in which we used the same pointing task and the same perceptual tasks, but in which the perceptual trials were interleaved with the pointing trials. Presenting the perceptual trials as a separate block in the present experiment may have defined them as a separate context, leading to less transfer.

A third factor that may affect the realignment is time: proprioceptive realignment has been found to occur slower compared to reach adaptation (Zbib *et al*., 2016). Consistently, found that the proprioceptive realignment continued to increase when the adaptation of motor responses had reached plateau (Fig. 3). This suggests that the realignment of the senses occurs on an even longer timescale than the slow component of error‐based adaptation of pointing responses.

Finally, cue conflict in rendered scenes may affect the reliability of the visual information and thereby the tendency to re‐align the proprioceptive sense to the visual sense. In our experiment, cue conflict was small as stereo disparity, motion parallax, shading and perspective all indicated the same distance. However, projecting the stimuli onto the same screen may have created conflict between accommodative cues and all other cues. Although this does only affect the reliability of information about depth and not the azimuth that we manipulated, we cannot exclude that this may have reduced proprioceptive realignment in comparison to a natural stimulus.

## Conclusion

In sum, our main finding is that temporally stable adaptation saturates rapidly, but is incomplete, and from that point on is robust over episodes as long as a week. This adaptation appears very specific: it did not transfer to the other hand and was not directly related to the perceptual estimates. The specificity of adaptation may be necessary to protect the adaptation from interference in other contexts: if the adaptation were not specific, natural visual feedback in between adaptation sessions would have interfered with the adaptation to the experimental perturbation. Thus, our data support the dual‐rate state space model and also show the context specificity of the model parameters 24 h of natural visual feedback barely interfered with the learning of the exposed perturbation, whereas interleaved episodes of adapting to a different perturbation are known to affect the subsequent adaptation (anterograde interference). A fascinating question for future research is whether performance in other types of task, for instance language acquisition, is similarly limited by a balance between learning and forgetting. Moreover, by discovering the factors that determine the retention and context specificity of learning, we may be able to develop targeted training protocols that enhance human performance.
